# Molecularly generated rat hepatitis E virus strains from human and rat show efficient replication in a human hepatoma cell line

**DOI:** 10.1016/j.virusres.2024.199364

**Published:** 2024-03-28

**Authors:** Jessica Panajotov, Alexander Falkenhagen, Ashish K. Gadicherla, Reimar Johne

**Affiliations:** a German Federal Institute for Risk Assessment, 10589 Berlin, Germany; b Center for Quantitative Cell Imaging, University of Wisconsin, Madison, USA

**Keywords:** Hepatitis E virus, Human, Rat, Zoonotic potential, Genomic clone, Hepatoma cell line

## Abstract

The hepatitis E virus (HEV) can cause acute and chronic hepatitis in humans. Whereas HEV genotypes 1–4 of species *Paslahepevirus balayani* are commonly found in humans, infections with ratHEV (species *Rocahepevirus ratti)* were previously considered to be restricted to rats. However, several cases of human ratHEV infections have been described recently. To investigate the zoonotic potential of this virus, a genomic clone was constructed here based on sequence data of ratHEV strain pt2, originally identified in a human patient with acute hepatitis from Hongkong. For comparison, genomic clones of ratHEV strain R63 from a rat and of HEV genotype 3 strain 47832mc from a human patient were used. After transfection of *in vitro*-transcribed RNA from the genomic clones into the human hepatoma cell line HuH-7-Lunet BLR, virus replication was shown for all strains by increasing genome copy numbers in cell culture supernatants. These cells developed persistent virus infections, and virus particles in the culture supernatant as well as viral antigen within the cells were demonstrated. All three generated virus strains successfully infected fresh HuH-7-Lunet BLR cells. In contrast, the human hepatoma cell lines HuH-7 and PLC/PRF/5 could only be infected with the genotype 3 strain and to a lesser extent with ratHEV strain R63. Infection of the rat-derived hepatoma cell lines clone 9, MH1C1 and H-4-II-E did not result in efficient virus replication for either strain. The results indicate that ratHEV strains from rats and humans can infect human hepatoma cells. The replication efficiency is strongly dependent on the cell line and virus strain. The investigated rat hepatoma cell lines could not be infected and other rat-derived cells should be tested in future to identify permissive cell lines from rats. The developed genomic clone can represent a useful tool for future research investigating pathogenicity and zoonotic potential of ratHEV.

## Highlights

• Infectious ratHEV was generated based on sequence data of a human patient strain.
• Comparison with HEV-GT3 and rat-derived ratHEV replication in different cell lines.
• All strains replicated efficiently in the human hepatoma cell line HuH-7-Lunet BLR.
• The results underline the zoonotic potential of ratHEV.
• The genomic clone can be used for further investigation on the emerging ratHEV.

## 1 Introduction

The hepatitis E virus (HEV) is one of the leading causes of acute hepatitis in humans worldwide, leading to estimated 3.3 million cases and 44,000 deaths per year (World Health Organisation). In addition, chronic hepatitis cases are increasingly recognized in immunosuppressed patients after infection with HEV genotype 3 (Velavan et al., 2021).

The HEV particle is non-enveloped with a diameter of 30 nm in stool samples. In addition, quasi-enveloped particles with a diameter of 40 nm are present in serum and cell culture supernatant. Both particle types are infectious in cell culture (Yin et al., 2016b).The HEV genome is a single-stranded RNA molecule of approximately 7 kb which encodes a non-structural polyprotein, a capsid protein and a small phosphoprotein (Pallerla et al., 2020).

HEV is classified into the family *Hepeviridae.* Within this family, the genus *Paslahepevirus* contains the main human-infecting genotypes 1–4. Genotypes 1 and 2 are only infectious for humans and are mainly transmitted by contaminated drinking water (Pallerla et al., 2020). In contrast, genotypes 3 and 4 are zoonotic and have a main reservoir in pigs. Their predominant transmission route to humans is via consumption of undercooked pork meat products (Velavan et al., 2021).

Another genus designated as *Rocahepevirus* contains the ratHEV, which mainly infects rats and is only distantly related to the HEV genotypes 1–4 (Johne et al., 2010a). RatHEV was first identified in 2010 in Norway rats from Germany (Johne et al., 2010b), but subsequently detected in different rat species from many countries worldwide (Reuter et al., 2020). First risk assessments suggested only a low potential of ratHEV for its transmission to humans and an initial infection trial with rhesus monkeys was not successful (Purcell et al., 2011). First serological evidence for human infections came from detection of ratHEV-specific antibodies in forestry workers in Germany (Dremsek et al., 2012) and in febrile patients in Vietnam (Shimizu et al., 2016). Beginning with 2018, ratHEV infections in human hepatitis cases have been reported from Hongkong, Canada, Spain and France (Sridhar et al., 2018, Andonov et al., 2019, Rivero-Juarez et al., 2022, Rodriguez et al., 2023). Clinical symptoms of ratHEV infection are similiar to those of genotype 1–4 (Sridhar et al., 2018). Recently, ratHEV infection could also be successfully reproduced in rhesus monkeys (Yang et al., 2022).

A few studies investigated the cell culture propagation and the generation of genomic clones for ratHEV to enable detailed laboratory analyses of this virus (Li et al., 2015, Chew et al., 2022, Fu et al., 2019). For instance, a genomic clone infectious for laboratory rats has been constructed for the ratHEV strain R63, originally derived from a wild Norway rat from Germany (Li et al., 2015)**.** Recently, Schemmerer et al. (2022) showed that this strain can be efficiently propagated in the human hepatoma cell line HuH-7-Lunet BLR. Limited cell culture propagation was also shown recently after inoculation of a ratHEV-containing stool sample from a human patient onto PLC/PRF/5 cells (Chew et al., 2022). However, no efficient cell culture system or infectious genomic clone has been established so far for a human-derived ratHEV strain, thus hindering the research on zoonotic potential of this virus.

Here, a molecular clone for the ratHEV strain pt2 originating from a human patient with acute hepatitis in Hongkong was constructed and compared to that of rat-derived ratHEV and human genotype 3 strains. The generation of replicating and infectious virus after transfection of *in vitro*-transcribed RNA into human hepatoma cell lines was analyzed by detection of genome, antigen and virus particles, as well as its infection potential by passaging the supernatant on cell cultures. Finally, human- and rat-derived hepatoma cell lines were tested for their ability to be infected with the viruses. The results indicate that infectious ratHEV strain pt2 can be generated from the molecular clone. The strain replicated efficiently in human hepatoma HuH-7-Lunet BLR cells, in accordance to its ability to cause hepatitis in a human patient. The molecular clone can therefore represent a useful tool for future research to investigate pathogenicity and zoonotic potential of ratHEV.

## 2 Material and methods

### 2.1 Cell lines

The human hepatoma cell line HuH-7 was kindly provided by Christian Drosten (Charité, Institute of Virology, Berlin, Germany) and maintained in Dulbecco's Minimum Essential Medium (DMEM) supplemented with 10 % fetal calf serum (FCS), 2 mM L-glutamine, 1 % non-essential amino acids (NEAA) and 1 % penicillin/streptomycin (10,000 U/ml, 10 mg/ml). All cell culture reagents and media were purchased from Pan Biotech (Aidenbach, Germany). The cell line HuH-7-Lunet BLR is a subclone of HuH-7 cells (Friebe et al., 2005), which was kindly provided by Mathias Schemmerer (University Medical Center Regensburg, Germany). This cell line and the human hepatoma cell line PLC/PRF/5 (ATCC CRL-8024, LGC Standards, Wesel, Germany) were maintained in Eagle's Minimum Essential Medium (MEM) supplemented with 10 % FCS, 2 mM L-glutamine, 1 % NEAA and 1 % penicillin/streptomycin. The cell line clone 9 (ATCC CRL-1439, LGC Standards) originating from a liver of Norway rat (*Rattus norvegicus*) and the Norway rat hepatoma cell line H-4-II-E (CCLV-RIE0224, kindly provided by Matthias Lenk, Friedrich Loeffler Institute, Germany) was cultivated in F12K Medium supplemented with 10 % FCS and 1 % gentamicin (10 mg/ml). The Norway rat hepatoma cell line MH1C1 (ATCC CCL-144, LGC Standards) was cultivated in a medium as described for H-4-II-E cells, but with 15 % horse serum. All six cell lines were maintained at 37 °C and 5 % CO_2_.

### 2.2 *Generation of genomic plasmids and* in vitro*-transcribed RNA*

Genomic plasmids for HEV genotype 3 strain 47832mc (Scholz et al., 2020a) and for ratHEV strain R63 (Li et al., 2015) have been described previously. For ratHEV strain pt2 (Sridhar et al., 2021), the complete genome sequence was derived from the GenBank database (accession number MN450851). By comparison of this sequence to other ratHEV strains, an unusual point-mutation was evident in the ORF2, which was considered as sequencing error and reverted (A3979G) for construction of the genomic plasmid. Three plasmids containing overlapping fragments of the pt2 genome were synthetized (IDT, Coralville, Iowa, USA), and a genomic plasmid containing the full-length genome sequence of strain pt2 flanked by a 5’-T7 RNA polymerase promoter and a 3’-unique restriction enzyme site in the vector pUCIDT‐Amp was constructed by standard molecular cloning methods (Scholz et al., 2020a). The correct sequence of the generated plasmid was confirmed by next generation sequencing as described previously (Scholz et al., 2020a). All genomic plasmids where linearized with respective restriction enzymes (BglII, XbaI and SwaI for the genomic plasmids of pt2, R63 and 47832mc, respectively) and cleaned up using the Monarch PCR & DNA Clean up kit (New England Biolabs GmbH, Frankfurt am Main, Germany). Thereafter, the plasmids were *in vitro-*transcribed using the MEGAscript T7 kit (Thermo Fisher Scientific, Waltham, MA, USA), treated with Turbo-DNAse (Thermo Fisher Scientific) and cleaned up using LiCl (Thermo Fisher Scientific) according to the manufacturer's instructions. The RNA was capped using the Vaccinia capping system with mRNA 2’-O-Methyltransferase (New England Biolabs GmbH, Frankfurt/Main, Germany) followed by an additional clean up using LiCl (Thermo Fisher Scientific). RNA was analyzed by electrophoresis on ethidium bromide-stained 1 % agarose gels.

### 2.3 Transfection of cell cultures

PLC/PRF/5, HuH-7 and HuH-7-Lunet BLR cells were seeded at a density of 7*10^5^ cells/T25 flask. PLC/PRF/5 and HuH-7-Lunet BLR were maintained in MEM supplemented with 10 % FCS, 2 mM L-glutamine, 1 % NEAA and 1 % penicillin/streptomycin. HuH-7 cells were cultured in DMEM supplemented as above. All cells were incubated at 37 °C and 5 % CO_2_. On the next day, cells showed confluency of 70 % and were transfected with *in vitro-*transcribed and capped RNA from the genomic plasmids using the *Trans*IT®-mRNA Transfection kit (Mirus Bio LLC, Madison, WI, USA). Equal amounts of transfection reagents and 7.5 µg RNA were mixed in 750 µl OptiMEM and added dropwise to flask with culture medium. One day later, the medium was completely removed and replaced by 5 ml fresh culture medium supplemented with 2.5 µg/ml amphotericin B and 30 mM MgCl_2_. Subsequently, the cells were incubated at 34.5 °C as described previously (Schemmerer et al., 2019)**.** The media were completely substituted with 5 ml fresh media twice weekly and supernatants were stored at −80 °C.

### 2.4 Infection of cell cultures

The conditions for infection of cell cultures were mainly based on the methods described for ratHEV strain R63 (Schemmerer et al., 2022) or HEV genotype 3 strains (Schemmerer et al., 2019). Briefly, cells were seeded at a density of 2.5*10^6^ cells/T25 flask 14 days prior to infection and maintained in 5 ml culture media as described above, with complete media exchanges twice weekly. Thereafter, media were removed and cells were washed twice with 2.5 ml phosphate-buffered saline (PBS). Cell culture supernatant from persistently infected HuH-7-Lunet BLR cells containing approximately 1 × 10^7^ HEV genome copies was mixed with MEM media (total volume 1000 µl) and added to the washed cells. After the cells were incubated at room temperature for 1 h, MEM supplemented with 10 % FCS, 2 mM L-glutamine, 1 % NEAA, 1 % penicillin/streptomycin, 2.5 µg/ml amphotericin B and 30 mM MgCl_2_ was added and cultures were maintained at 34.5 °C and 5 % CO_2_. The media were completely exchanged twice weekly and supernatants were stored at −80 °C until further analysis.

### 2.5 RT-qPCR

RNA was extracted from 450 µl cell culture supernatant and eluted in 60 µl using the EMAG Nucleic Acid Extraction System (Biomeriéux Deutschland GmbH, Nürtingen, Germany). RT-qPCR for detection of HEV genotype 3 was performed using the primer/probe system of Jothikumar et al. (2006) under conditions as described by Schielke et al. (2011). For detection of ratHEV (both strains), primer HEV-C-S (5’-CTTgTTgAgCTYTTCTCCCCT-3’), primer HEV-C-A (5’-TgTACCggATgCgACCAA-3’) and probe HEV-C-TM2 (6FAM-5’-TGCAgCTTgTCTTTgARCCCgC-3’-BBQ) (Sridhar et al., 2021, slightly modified by Sally Baylis, Paul-Ehrlich-Institute, Germany, and Marco Kaiser, TIB-Molbiol, Germany) were used. The final master mix contained 10 µl 2x Quantitect probe RT-PCR Master Mix, 0.2 µl Enzyme RT-Mix, 8 pmol of each primer and 4 pmol probe in a total volume of 15 µl. Temperature conditions where the same as described by Schielke et al. (2011). The *in vitro*-transcribed RNAs from the genomic plasmids (see 2.2) were used as standards for quantification.

### 2.6 Immunofluorescence assay

Persistently infected HuH-7-Lunet BLR cells were seeded into a 96-well plate at a density of 10^4^ cells/well. Fourteen days post-seeding, cells were fixed using 1:1 acetone/methanol for 1h at 4 °C. A mixture of 1 % FCS in PBS was prepared for blocking and to dilute primary and secondary antibodies. A total of 50 µl/well of a 1:100 dilution (original concentration 1.2 mg/ml) of the mouse monoclonal antibody 9C8 (kindly provided by Aurelija Zvirbliene, Vilnius University Life Sciences Center, Institute of Biotechnology, Lithuania) was added to fixed cells. This antibody was originally elicited against the capsid protein of HEV genotype 3, but has previously been shown to cross-react with ratHEV (Simanavicius et al., 2018). After 1 h at 37 °C, cells were washed three times with PBS and 50 µl of goat anti-mouse IgG (whole molecule)-FITC (Sigma, Deisenhofen, Germany) was added at a dilution of 1:200. After an additional 1 h incubation at 37 °C, the plate was washed twice with PBS and once with distilled water. The cells were mounted with Roti®-Mount Fluor Care DAPI (Carl Roth, Karlsruhe, Germany) and analyzed using an Axio Observer Z1 microscope (Carl Zeiss, Oberkochen, Germany).

### 2.7 Electron microscopy

Cell culture supernatant was adsorbed onto 400 mesh carbon-formvar coated copper grids (Plano GmbH, Germany) for 5 min followed by fixation with 2.5 % glutaraldehyde for 1 min. Excess liquid was removed using tissue paper and the grids were contrasted with 2 % uranyl acetate for 1 min. After removal of excessive liquid, the grids were dried and examined in a Jeol 1400 Plus TEM (Jeol, Japan), operated at 120 kV. The particle diameter was measured using ITEM software (Olympus, Germany).

### 2.8 Next generation sequencing (NGS) and sequence analysis

Sequencing of the genome of strain R63 generated after transfection of HuH-7-Lunet BLR cells was performed by NGS as described previously (Scholz et al., 2020a). The genome sequence analyses were done using the ClustalW method with the MegAlign Pro 17 module of the DNASTAR software (Lasergene, Madison, WI, USA).

## 3 Results

### 3.1 Establishment of a genomic clone for ratHEV strain pt2

**Fig. 1:**
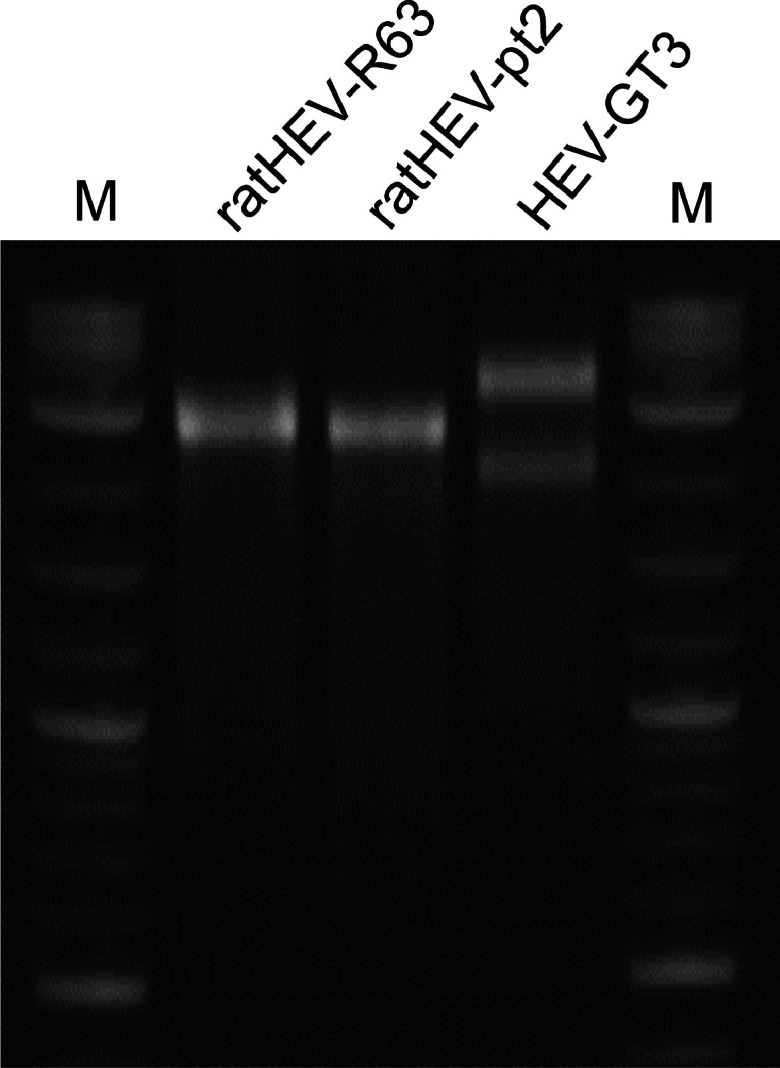
Analysis of *in vitro*-transcribed and capped RNA of ratHEV strain R63, ratHEV strain pt2 and HEV genotype 3 (GT3) strain 47832mc after electrophoresis on an ethidium bromide-stained agarose gel. M: Quick Load 1kb Plus DNA Ladder (New England Biolabs GmbH).

A plasmid containing the whole genome sequence of the human-derived ratHEV strain pt2 (GenBank acc.-no. MN450851 with mutation A3979G, see chapter 2.2) under transcriptional control of the T7 RNA polymerase promoter was generated from synthetized DNA sequence fragments. A genome alignment showed that ratHEV strain pt2 had nucleotide sequence identities of 79 % with the rat-derived ratHEV strain R63 and 61 % with the human-derived HEV genotype 3 strain 47832mc. Genomic RNA was *in vitro*-transcribed and capped from the genomic plasmid of strain pt2 as well as from the available plasmids containing the genome sequences of strains R63 and 47832mc. Analysis of the RNAs by electrophoresis on an ethidium bromide-strained agarose gel indicated a band of the expected length for pt2 and R63, whereas 47832mc produced 2 bands as already described (Scholz et al., 2020a) for unknown reasons (Fig. 1).

### 3.2 Virus replication after transfection of genomic RNA into human hepatoma cell lines

**Fig. 2:**
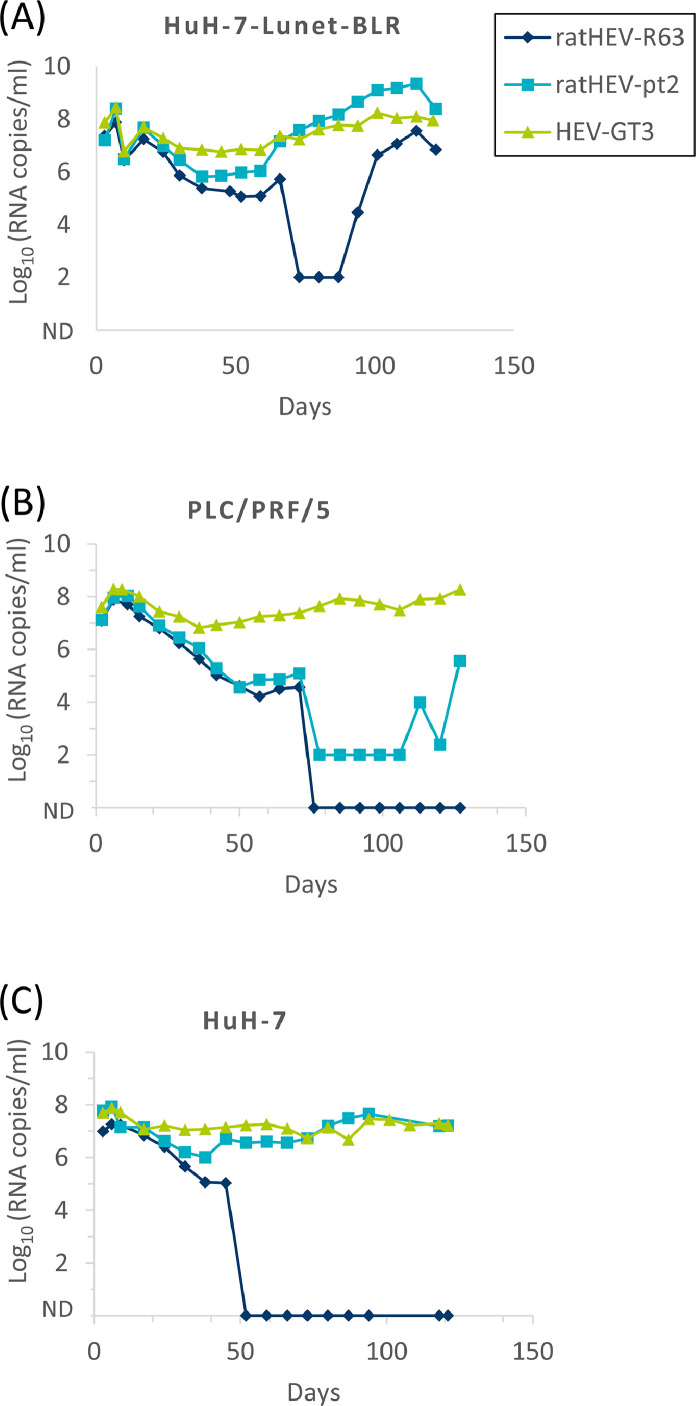
Detection of viral RNA in the supernatant of cell cultures after transfection with *in vitro*-transcribed and capped viral RNA. The human hepatoma cell lines HuH-7-Lunet BLR (A), PLC/PRF/5 (B) and HuH-7 (C) were transfected with RNA of ratHEV strains R63 or pt2, or HEV genotype 3 (GT3) strain 47832mc and analysed by RT-qPCR at the indicated time-points. ND: not detectable.

The human hepatoma cell lines PLC/PRF/5, HuH-7 and HuH-7-Lunet BLR were transfected with the *in vitro*-transcribed and capped genomic RNAs, cultivated for 120 days, and culture supernatants were screened weekly by RT-qPCR (Fig. 2). For HEV genotype 3 strain 47832mc, relatively constant genome quantities of about 10^8^ genome copies/ml were detected over the whole time period in all three cell lines. RatHEV strain pt2 also showed about 10^8^ genome copies/ml in HuH-7 and HuH-7-Lunet BLR cells over the course of the experiment. However, in PLC/PRF/5 cells, genome copies slowly decreased during the first weeks of the experiment followed by an increase beginning at day 100. The number of ratHEV strain R63 genome copies decreased to undetectable levels in HuH-7 and PLC/PRF/5 cells. In HuH-7-Lunet BLR cells, the genome copy numbers of this strain decreased at day 73, but increased after day 87 reaching 10^7^ genome copies/ml at day 120. To check for potential mutations which might have occurred in this strain after day 87, culture supernatant from day 101 was subjected to genome sequencing by NGS. However, comparison of this genome sequence to that of the original plasmid did not identify any mutation.

### 3.3 Characterization of persistently infected HuH-7-Lunet BLR cells

**Fig. 3:**
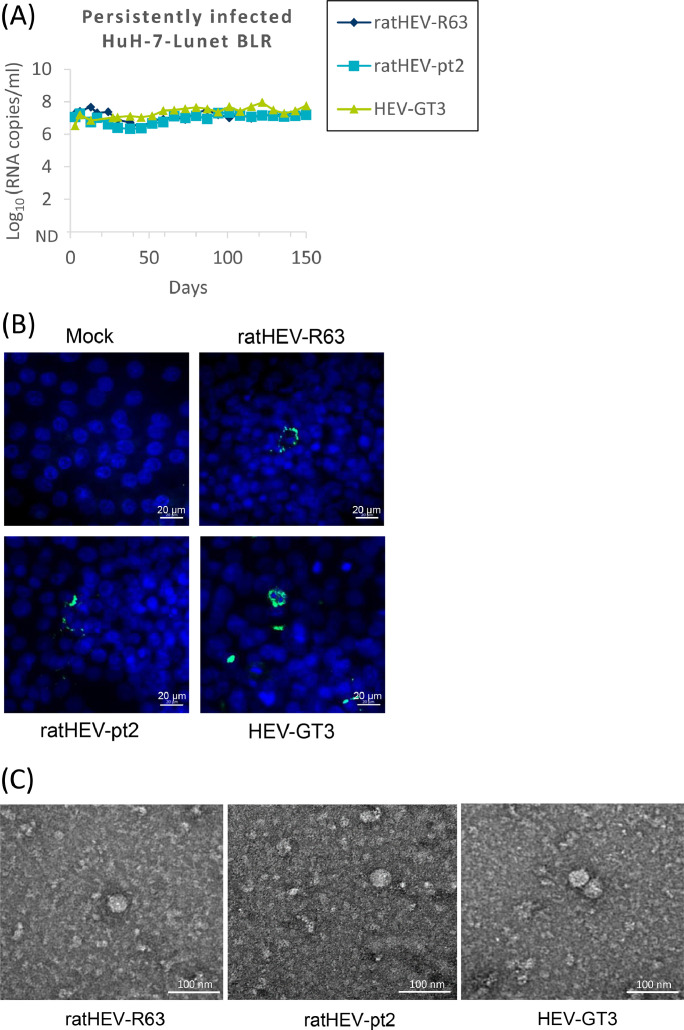
Characterization of HuH-7-Lunet BLR cells persistently infected with ratHEV strains R63 or pt2, or HEV genotype 3 (GT3) strain 47832mc. The cells were generated by splitting at 120 days after transfection with the respective genomic RNA. (A) Detection of viral RNA. The culture supernatant was analysed by RT-qPCR at the indicated time-points. ND: not detectable. (B) Detection of viral antigen. The cells were stained with the monoclonal antibody 9C8 directed against the HEV capsid protein and visualized by immunofluorescence (green). Cellular nuclei were stained with DAPI (blue). Scale bar: 20 µm. (C) Detection of viral particles. The culture supernatant was analyzed by transmission electron microscopy after negative staining with uranyl acetate. Scale bar: 100 nm.

As all virus strains seemed to replicate in HuH-7-Lunet BLR cells, the transfected cells were split at day 120 and one flask for each virus was further cultivated for 150 days. RT-qPCR analysis indicated that all of the virus strains constantly produced virus as demonstrated by detection of 10^7^–10^8^ genome copies/ml culture supernatant over the whole time period (Fig. 3A). Intracellular production of viral antigen was analyzed by immunofluorescence staining using the monoclonal antibody 9C8 that was raised against the genotype 3 capsid antigen and shown to cross-react with ratHEV (Simanavicius et al., 2018). A granular perinuclear fluorescence typical for the HEV capsid protein could be demonstrated in some of the cells for all virus strains (Fig. 3B). Furthermore, the culture supernatants were subjected to electron microscopy and spherical particles with a diameter of approximately 40 nm were identified for all strains (Fig. 3C).

### 3.4 Virus replication after infection of human and rat hepatoma cell lines

**Fig. 4:**
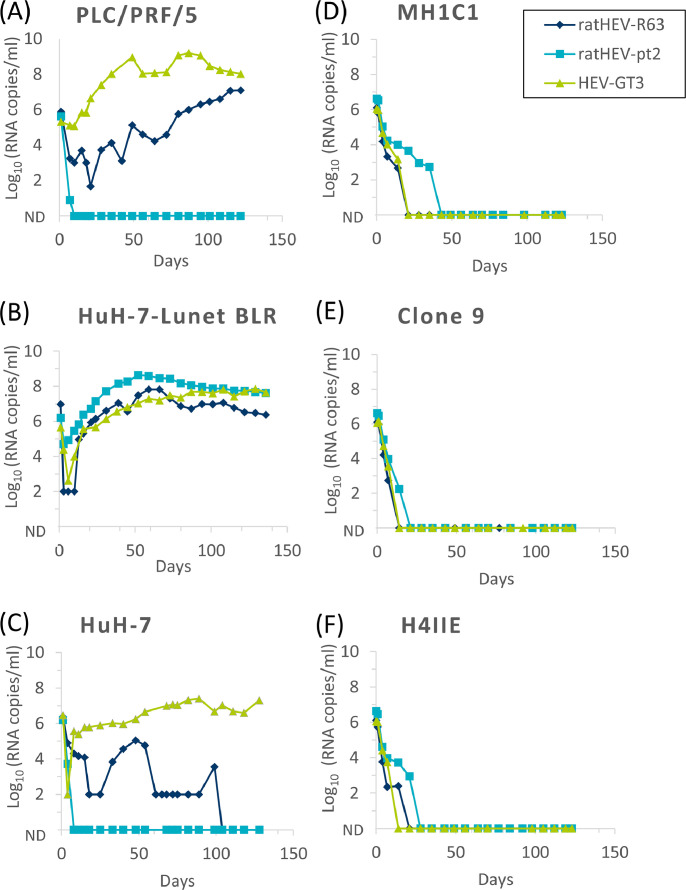
Detection of viral RNA in the supernatant of cell cultures after infection with ratHEV strains R63 or pt2, or HEV genotype 3 (GT3) strain 47832mc. The human hepatoma cell lines PLC/PRF/5 (A), HuH-7-Lunet BLR (B) and HuH-7 (C) and the rat hepatoma cell lines MH1C1 (D), clone 9 (E) and H-4-II-E (F) were inoculated with supernatant from persistently infected HuH-7-Lunet BLR cells and analysed by RT-qPCR at the indicated time-points. ND: not detectable.

In order to demonstrate the presence of infectious virus and to compare replication kinetics, the culture supernatants of the persistently infected HuH-7-Lunet BLR cells were inoculated onto the three human hepatoma cell lines HuH-7-Lunet BLR, HuH-7 and PLC/PRF/5 cells (Fig. 4, left) as well as the rat hepatoma cell lines clone 9, MH1C1 and H-4-II-E (Fig. 4, right) using equal genome copy numbers. Cells were cultivated for 120 days and supernatants were screened by RT-qPCR as before. As shown in Fig. 4, all virus strains showed increasing genome amounts in HuH-7-Lunet BLR cells reaching 10^8^ genome copies/ml for strains 47832mc and pt2, and 10^7^ genome copies/ml for strain R63. In contrast, only strain 47832mc replicated efficiently in HuH-7 cells, whereas strain pt2 was rapidly undetectable and R63 was only detectable until day 100. In PLC/PRF/5 cells, strains 47832mc and R63 were continuously detectable, while strain pt2 did not show evidence of replication. No efficient replication could be demonstrated for either strain in the three rat-derived hepatoma cell lines.

## 4 Discussion

Human cases of ratHEV infection have been increasingly recognized during the last years (Sridhar et al., 2018, Andonov et al., 2019, Rivero-Juarez et al., 2022, Rodriguez et al., 2023, Sridhar et al., 2021). However, research on this virus was hampered by the lack of efficient cell culture methods and reverse genetics systems for human-derived ratHEV strains. Here, we successfully generated infectious virus of ratHEV strain pt2 originally detected in a human hepatitis patient from Hongkong, and efficient replication in cell culture was demonstrated. The molecular clone of the virus was directly generated from published sequence data, which might represent a universal approach that could be applied to other HEV strains in future.

The most suitable cell line for all strains tested here was HuH-7-Lunet BLR. This cell line was originally generated by infection with hepatitis C virus and subsequent curation of this infection (Friebe et al., 2005). It is highly susceptible to hepatitis C virus, but has recently also been shown to efficiently replicate HEV genotype 3 and the ratHEV strain R63 (Schemmerer et al., 2022, Schemmerer et al., 2019). We showed here that the human-derived ratHEV strain pt2 can also be well propagated in this cell line. The virus reached titers of 10^8^ genome copies/ml in the culture supernatant of transfected or infected HuH-7-Lunet BLR cells. In contrast, only limited replication was found in PLC/PRF/5 cells, confirming findings of another study, in which PLC/PRF/5 cells were inoculated with a human stool sample containing ratHEV (Chew et al., 2022).

The generated virus was further characterized. Virus antigen could be demonstrated in infected HuH-7-Lunet BLR cells using immunofluorescence, which showed the typical perinuclear distribution of the HEV capsid protein (Simanavicius et al., 2018)**.** However, the antibody was originally raised against genotype 3 and it is unclear how well ratHEV capsid protein is detected; therefore, the future development of ratHEV-specific antibodies and comparisons of antibody specificity would be beneficial. Electron microscopy mainly identified particles with a diameter of approximately 40 nm in the supernatant. They were similar to quasi-enveloped HEV particles, which are usually found in culture supernatant (Yin et al., 2016a). The presence of infectious virus in the supernatant was also demonstrated by successful infection of fresh HuH-7-Lunet BLR cells, which resulted in high virus titers.

Infection of the different human hepatoma cell lines with the generated viruses led to different results. Whereas the genotype 3 strain replicated well in all of these cells, the replication of the ratHEV strains was less efficient in HuH-7 and PLC/PRF/5 cells. As they generally replicated better in those cell lines after transfection of their genomic RNA, a reduced infectivity of the used inoculum might be responsible. HEV from culture supernatant is mostly quasi-enveloped and generally less infectious than non-enveloped HEV (Yin et al., 2016a, Yamada et al., 2009, Ji et al., 2021). In addition, cellular proteins can be present in the quasi-envelope (Oechslin et al., 2020), which might contribute to a cell type-specific infectivity of these virus particles. It could also be that so far unknown cellular receptor molecules for the ratHEV strains are missing on those cell lines, but are present on HuH-7-Lunet BLR cells.

Inoculation onto rat hepatoma cell lines did not result in replication of ratHEV or genotype 3 strains. Previous studies also showed the lack of significant replication of ratHEV after inoculation of these cell lines with rat-derived samples (Johne et al., 2010a, Debing et al., 2016), despite a tropism of ratHEV to the liver in infected rats (Johne et al., 2010a, Zhang et al., 2023)**.** The distinct reasons are not known, but the used hepatoma cell lines might have a too distinct phenotype to be infectible by the virus. Transfection experiments of the cell lines with *in vitro*-transcribed genomic RNA of the HEV strains should be performed in future to analyse if virus entry or other steps of the viral life cycle are blocked in these cell lines. In addition, further testing of different rat-derived cells will be necessary to establish a suitable *in vitro* system for testing ratHEV replication in rat cells.

## 5 Conclusions

We demonstrated the successful generation of an infectious molecular clone of a human-derived ratHEV strain. An effective cell culture system suitable for analysis of the complete replication cycle of different ratHEV strains as well as HEV genotype 3 strains was identified. Persistently infected cells have been generated, which enable continuous virus production. Using the molecular clone, targeted mutations can be introduced into the human-derived ratHEV enabling reverse genetics research approaches as already available for some other HEV strains (Scholz et al., 2020b). Successful infections of human hepatoma cells of human- and rat-derived ratHEV strains underline the zoonotic potential of ratHEV. The tools developed here might help to further assess the pathogenic and zoonotic properties of different ratHEV strains and enable more detailed research on this novel emerging virus in future.

### Funding acknowledgement

This study was funded by the German Federal Ministry of Education and Research (Project ZoRaHED, grant number 01KI2103).

### Data availability

Further data are available upon request from R.J.

## CRediT authorship contribution statement

**Jessica Panajotov:** Formal analysis, Investigation, Methodology, Writing – original draft. **Alexander Falkenhagen:** Data curation, Investigation, Methodology, Writing – review & editing. **Ashish K. Gadicherla:** Investigation, Methodology, Writing – review & editing. **Reimar Johne:** Conceptualization, Funding acquisition, Project administration, Supervision, Writing – review & editing.

## Declaration of competing interest

The authors declare that they have no known competing financial interests or personal relationships that could have appeared to influence the work reported in this paper.

## Data Availability

Data will be made available on request.
